# Hand hygiene instruction decreases illness-related absenteeism in elementary schools: a prospective cohort study

**DOI:** 10.1186/1471-2431-12-52

**Published:** 2012-05-15

**Authors:** Claudia H Lau, Elizabeth E Springston, Min-Woong Sohn, Iyana Mason, Emily Gadola, Maureen Damitz, Ruchi S Gupta

**Affiliations:** 1 Smith Child Health Research Program, Children’s Memorial Hospital, 2300 Children’s Ave, Box 157, Chicago, IL 60614, USA; 2 Institute for Healthcare Studies, Northwestern University Feinberg School of Medicine, 750 N Lake Shore Drive, 10th Fl, Chicago, IL 60611, USA; 3 Center for Management of Complex Chronic Case, Hines VA Hospital, 5000 South 5th Avenue, Hines, IL 60141, USA; 4 Stakeholders Collaboration to Improve Student Health, Respiratory Health Association of Metropolitan Chicago, 1440 W. Washington Blvd., Chicago, IL 60607, USA

**Keywords:** Hand hygiene, Education, Elementary school, Illness

## Abstract

**Background:**

Illness-related absences have been shown to lead to negative educational and economic outcomes. Both hand washing and hand sanitizer interventions have been shown to be effective in reducing illness-related absences. However, while the importance of hand hygiene in schools is clear, the role of instruction in use is less obvious. The purpose of this study was to compare absenteeism rates among elementary students given access to hand hygiene facilities versus students given both access and short repetitive instruction in use, particularly during influenza season when illness-related absences are at a peak.

**Methods:**

A hand hygiene intervention was implemented from October to May during the 2009/2010 academic year, including peak flu season, in two Chicago Public Elementary Schools among students grades pre-kindergarten to eighth grade (ages 4–14). Classrooms were systematically assigned to an intervention or control group by grade (cluster design). Hand hygiene facilities (sanitizer and soap) were made available to all students. Students in the intervention group also received short repetitive instruction in hand hygiene every 2 months. Only absences as a result of respiratory or gastrointestinal illness were used to establish illness-related absenteeism rates. Percent absent days were calculated and bivariate analyses were performed to compare percent absent days among students given access to hand hygiene facilities versus students given both access and instruction. Prior to the intervention, teachers’ perceptions of students’ hand hygiene were also evaluated. Teacher perceptions were analysed to describe attitudes and beliefs.

**Results:**

Data were collected and analysed for 773 students reporting 1,886 absences during the study period (1.73% of total school days). Both the percent total absent days and percent illness-related absent days were significantly lower in the group receiving short instruction during flu season (P = 0.002, P < 0.001, respectively). This difference peaked during the influenza season (when intervention began) and declined in the following months. Teachers (n = 23) agreed that hand hygiene is not performed properly among students and reported time constraints as a barrier to frequent hand washing.

**Conclusions:**

Adding hand hygiene instruction to existing hand hygiene practices improved attendance at public elementary schools during the flu season. Standardized and brief repetitive instruction in hand hygiene holds potential to significantly reduce absenteeism.

## Background

Absenteeism is a major problem among school-aged children, with approximately 75% of all school absences attributed to illness [1,2]. Illness-related absences have been shown to lead to negative educational and economic outcomes. For example, a sick child may fall behind in his or her coursework and suffer academically [3,4]. Teacher absences due to exposure may delay learning in the classroom [5,6]. Finally, schools may lose public funding due to absenteeism [7].

As hands are an important mode of transmission of infectious disease among school-aged children, hand hygiene is critical in reducing illness-related absences [8]. Both hand washing and hand sanitizing have been shown effective to this end [9-14]. Hand washing interventions have been shown to significantly reduce illness-related absences in elementary school students by as much as 26% [9], and significantly reduce a subset of illness-related absences (i.e. gastrointestinal illnesses) by as much as 32% [10]. Hand sanitizers have been shown to be an effective alternative to conventional hand washing when hands are not visibly dirty, with one study showing a 51% decrease in illness-related absences compared to usual hand washing practices [11-15].

While the importance of hand hygiene among school-aged children is clear, the role of instruction in use is less obvious. It is unclear if absenteeism is further reduced when access to sink, soap, and hand sanitizer is coupled with regular instruction in hand hygiene. The purpose of this study was to compare absenteeism rates among elementary students given access to hand hygiene facilities versus students given both access and short repetitive instruction in use, particularly during influenza season when illness-related absences are at a peak.

## Results

### Participant characteristics

A total of 981 students were eligible and participated in the study; no students opted out or discontinued participation during the course of the study (Table 1). Data from grades pre-kindergarten and kindergarten were not used in analyses as a result of inconsistent attendance records, giving a final sample of 773 students. A total 1,913 absences were recorded for students in grades 1 through 8 during the study period. Twenty seven recorded absences were not used in analyses due to missing data (i.e., reason and date of absence), for a total of 1,886 data points.

**Table 1:** Demographic characteristics of study participants

**Variable**	**No. of Children (%)**			
	**Alcott**		**Walsh**	
	**(N = 502)**		**(N = 479)**	
Race/ethnicity				
White	271	(54.0)	0	(0.0)
Black	96	(19.1)	29	(6.0)
Hispanic/Latino	101	(20.1)	444	(92.7)
Others	34	(6.7)	6	(1.3)
Gender				
Male	215	(42.9)	246	(51.4)
Female	287	(57.1)	233	(48.6)
Grade				
Pre-Kindergarten (ages 4–5)	60	(12.0)	40	(8.4)
Kindergarten (ages 5–6)	61	(12.2)	47	(9.8)
First (ages 6–7)	56	(11.2)	39	(8.1)
Second (ages 7–8)	49	(9.8)	48	(10.0)
Third (ages 8–9)	48	(9.6)	55	(11.5)
Fourth (ages 9–10)	49	(9.8)	59	(12.3)
Fifth (ages 10–11)	47	(9.8)	49	(10.2)
Sixth (ages 11–12)	48	(9.6)	51	(10.6)
Seventh (ages 12–13)	46	(9.2)	34	(7.1)
Eighth (ages 13–14)	38	(7.6)	57	(11.9)
Low Income Students	157	(31.2)	448	(93.5)
Limited English Learners	32	(6.4)	102	(21.2)

### Absenteeism rates

**Figure 1:**
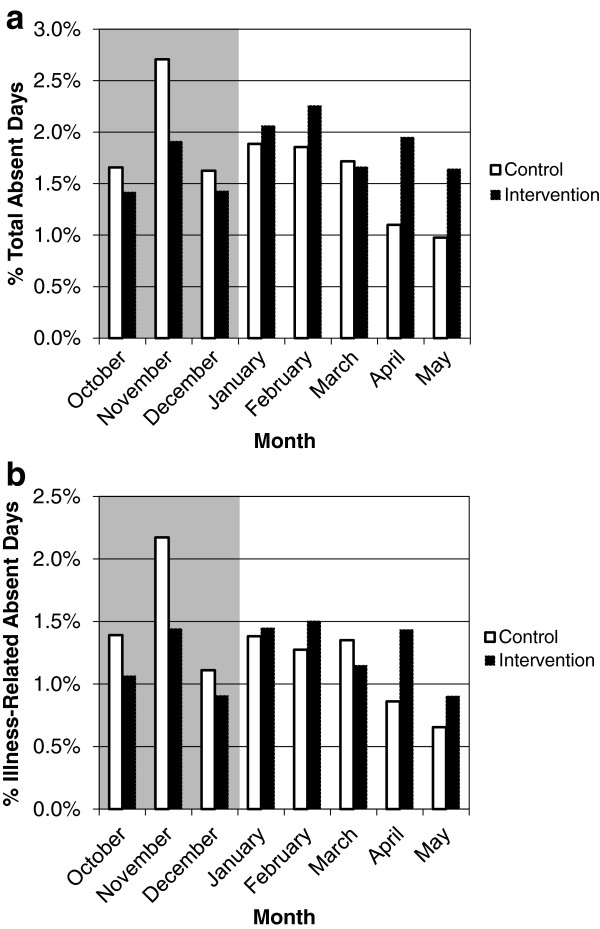
**a Percent total absent days during the 2009-2010 academic year. b** Percent illness-related absent days during the 2009-2010 academic year. Shaded area = influenza season.

Percent total absent days are listed in Table 2; percent illness-related absent days are listed in Table 3. Both the collapsed total rate and collapsed illness-related rate of absenteeism were significantly lower in the intervention groups during influenza season. This difference peaked during the influenza season (when intervention began) and declined in the following months (Figure 1a and b). The peak in percent absent days matched the peak in number of influenza-like illnesses in 2009 (both regular and pandemic i.e. H1N1) reported by the City of Chicago [16].

**Table 2:** Total absenteeism during the 2009-2010 academic year

	**Both schools**		**Alcott**		**Walsh**	
	**Control**	**Intervention**	**Contro**l	**Intervention**	**Control**	**Intervention**
**During the academic year (October to May)**						
Total absence days	879	1,007	539	519	340	488
Total possible days of attendance*	52,734	56,259	27,777	25,944	24,957	30,315
% total absent days	1.67	1.79	1.94	2.00	1.36^1^	1.61^1^
**During influenza season (October to December)**						
Total absence days	365	309	249	186	116	123
Total possible days of attendance*	18,326	19,551	9,653	9,016	8,673	10,535
% total absent days	1.99^2^	1.58^2^	2.58^1^	2.06^1^	1.34	1.17

*Total number of students multiplied by possible days of attendance.

^1^P<0.05

^2^P<0.01.

**Table 3:** Illness-related absenteeism during the 2009-2010 academic year

	**Both schools**		**Alcott**		**Walsh**	
	**Control**	**Intervention**	**Control**	**Intervention**	**Control**	**Intervention**
**During the academic year (October to May)**						
Illness-related absence days	655	692	450	411	215	281
Total possible days of attendance*	52,734	56,259	27,777	25,944	24,957	30,315
% illness-related absent days	1.26	1.23	1.62	1.58	0.86	0.93
**During influenza season (October to December)**						
Illness-related absence days	288	224	208	150	80	74
Total possible days of attendance*	18,326	19,551	9,653	9,016	8,673	10,535
% illness-related absent days	1.57^2^	1.15^2^	2.15^1^	1.66^1^	0.92	0.70

*Total number of students multiplied by possible days of attendance.

^1^P<0.05

^2^P<0.01.

### Teachers’ perceptions

Survey responses were received for 23 of 30 teachers in participating schools (Table 4). The majority of respondents agreed that students wash their hands during the school day, but did not believe that students do so properly. Narrative data suggest that students most often wash their hands after restroom breaks and before lunch. However, responses varied widely, with some teachers encouraging students to wash their hands as needed and others limiting hand washing to scheduled bathroom breaks. Both schools reported time constraints as a barrier to frequent hand washing.

**Table 4:** Itemized perceptions of hand hygiene among teachers prior to the intervention (n = 23)

**Item**	**Responses, % Yes**
***Students’ hand washing practices***	
Students wash hands with soap and water during the school day	82.6
Students in your classroom wash their hands properly	21.7
Barriers prevent you and your students from proper hand washing	50.0
***Students’ hand sanitizer practices***	
Students use hand sanitizer during the school day	91.3
Students in your classroom use hand sanitizer properly	56.5
***Relationship between hygiene and illness***	
Students’ poor hygiene habits impact the amount of illness in your classroom	56.5
Availability of hand sanitizer in your classroom would help decrease the amount of illness in your classroom	86.4

In response to the H1N1 outbreak of 2009, both schools were supplied with hand sanitizer by Chicago Public Schools prior to the beginning of this study [17]. Hand sanitizer use was reported to be commonplace in both schools, although half of teachers believed their students used hand sanitizer incorrectly. Walsh teachers observed that students only used hand sanitizer before meals and after recess while teachers at Alcott reported that most students used hand sanitizer as needed.

## Discussion

This study demonstrates that regular hand hygiene instruction may be useful in reducing illness-related absences during the flu season. Flu season is a critical time for attendance improvement, as illness-related absences are traditionally highest during these months [18,19]. Consistent with past research, our findings demonstrate the importance of instruction in improving efficacy of hand hygiene practices at schools [9-15]. As observed, interventions to change hygiene behaviour are plausible among children [20].

Data from teachers suggest that hand hygiene standards vary greatly from school to school, with one exception: hand hygiene is not performed properly among students. Additional barriers to hand hygiene were consistent with those reported in other studies and included time constraints and limited access to materials/facilities [21,22]. Such findings underscore the importance of compulsory instruction in hand washing and sanitizing techniques as well as uniform distribution and access policies.

We accordingly recommend a two-part hand hygiene policy in public elementary schools: (1) We advise schools to ensure that all common areas are well-stocked with hand sanitizer and that all bathrooms are well-stocked with hand washing materials throughout the school day. (2) We urge schools to provide a short hand hygiene lesson for students at the beginning of each academic year, as well as refresher lessons throughout the year.

This study is not without limitations. The sample was small and convenience-based, resulting in low statistical power. Small sample size may be the reason for correct directionality without statistical significance until results from both schools were analyzed as a whole. Significant differences detected in outcomes were likely conservative due to limitations in the study design: Per request of school administration, we use alcohol-free hand sanitizer rather than alcohol-based hand sanitizer. We also did not have data on influenza vaccination rates of children in the participating schools, and did not attempt to stop children in the intervention group from passing on hand hygiene instruction to children in the control group. Moreover, the intervention was conducted at a time of heightened hand hygiene awareness following the H1N1 outbreak, which likely resulted in more vigilance in hand hygiene among both control and intervention groups. Finally, our analysis did not correct for clustering at the class level and a simple *t*-test of absenteeism rates in the two groups at the cluster level (n = 31) did not show any significant associations due to lack of statistical power. Accordingly, our results need to be interpreted cautiously.

## Conclusions

The illness-related absenteeism rate decreased during influenza season when hand hygiene instruction was added to existing hand hygiene practices. As a result, we recommend implementation of standardized hand hygiene policies to ensure access to materials/facilities and regular instruction in use. The proposed policy may reduce loss of funding due to illness-related absenteeism, and, more importantly, reduce student illness and improve students’ academic performance.

## Methods

A hand hygiene intervention was implemented from October to May during the 2009/2010 school year in two Chicago Public Elementary Schools among students’ grades pre-kindergarten to 8. The study period included peak flu season, which occurs from October through December [23]. The study protocol was reviewed and approved by the Institutional Review Board of Children’s Memorial Hospital and the Chicago Public Schools Research Review Board in Chicago, Illinois.

### Participants

Eligible students were enrolled in grades pre-kindergarten through eighth (ages 4 –14) at Louisa May Alcott Elementary School or Walsh Math and Science Academy in Chicago, Illinois. Caregivers were given the opportunity to opt their child out of the intervention, with consent implicit in the absence of a signed opt-out form. The intervention was conducted with the consent and support of participating schools.

### Study design

Systematic sampling was used to assign odd grades to the intervention group (n = 15) and even grades to the control group (n = 16) (cluster design).

**Figure 2:**
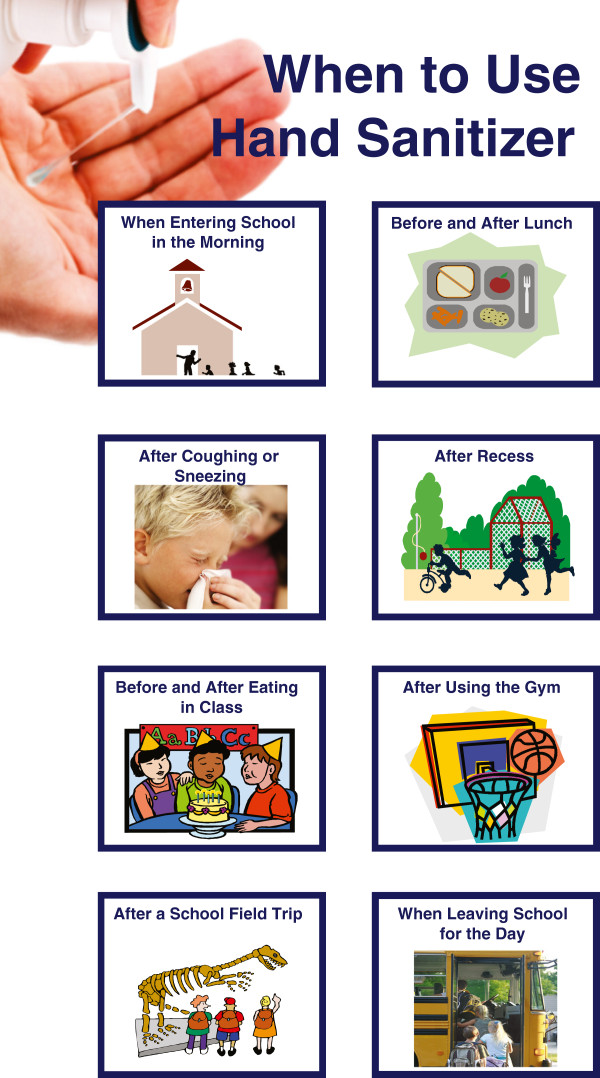
Hand sanitizer use protocol, distributed and implemented in all classrooms.

Hand sanitizer and hand washing facilities were made available to students in both the intervention and control group. Dispensers of alcohol-free hand sanitizer (active ingredient, 0.13% Benzalkonium Chloride) were mounted near the doorway inside every classroom and near entrances to common areas, including the main office, bathrooms, lunchrooms, computer laboratories, and gymnasiums. (Hand sanitizer was not placed in restrooms to avoid disruption of hand washing with soap and water.) Posters describing when to use the hand sanitizer were hung up throughout the schools (Figure 2). Children were instructed to use soap and water in lieu of hand sanitizer when hands were visibly dirty. Study personnel conducted weekly checks to ensure that hand sanitizer and soap were properly stocked and available to all students.

**Figure 3:**
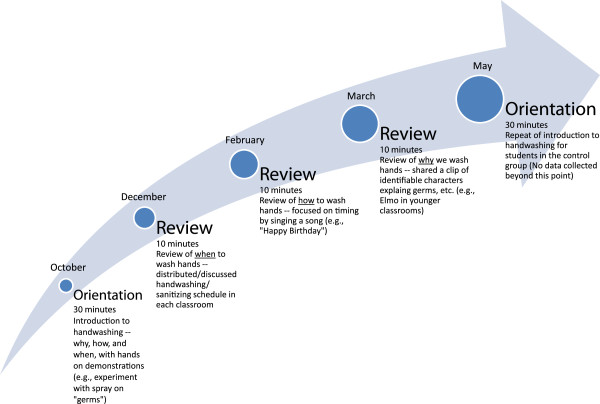
Overview of hand hygiene curriculum administered in intervention classrooms.

In addition to these measures, intervention classrooms were given a protocol for hand sanitizer use and received regular instruction in hand hygiene from study personnel. Grade appropriate curriculum was used to instruct students in proper hand washing with an emphasis on the importance of hand hygiene. The curriculum included an initial 30-minute interactive session, which used a black light experiment with glow-in-the-dark “germ” lotion, trivia games (grades 2 through 8), and a demonstration with finger puppets (pre-kindergarten and kindergarten), as well as three 10-minute review sessions every two months, focusing on when, how, and why to use hand hygiene (Figure 3). At the conclusion of the study, control classrooms also received the 30-minute lesson on hand hygiene. The instructions were all in English and all students had a good grasp on the English language. Teachers and instructors made sure all children participated in and understood the lessons.

### Statistical analysis

Per school policy, data were collected documenting each student absence as reported by parents. If reason for absence was not submitted, study personnel called parents to determine reason for absence. For the purposes of this study, study personnel classified a respiratory or gastrointestinal illness as an illness-related absence. Other illnesses or excuses were documented as non-illness-related. Per school policy, however, we were allowed to use class-level aggregate data on study absences. Prior to the intervention, teachers’ perceptions of students’ hand hygiene were also evaluated.

Percent total absent days was calculated as the ratio of total absence days to all possible days of attendance. Percent illness-related absent days was calculated as the ratio of illness-related absence days to all possible days of attendance, illness-related or otherwise. Rates were calculated for the overall academic year (October to May) and for influenza season (October to December) [23].

*χ*^2^ tests of independence were performed to determine whether the number of absent student-days (total and illness-related) differed significantly between intervention and control groups.

To describe teacher perceptions of student hand hygiene, a brief survey was administered prior to intervention and response categories were collapsed into yes/no. Response frequencies were calculated. Qualitative narrative data were also collected and are summarized in the results section.

All statistical analyses were performed using Stata/SE 10.0 (Stata Corp LP, College Station, TX) and Microsoft Excel 2010 (Microsoft Corp, Redmond, WA).

## Competing interests

The authors declare that they have no competing interests.

## Authors’ contributions

CHL and MWS performed the statistical analysis and drafted the manuscript. EES designed the study, carried out the intervention, and assisted with interpretation of data and critical review of the manuscript. IM, EG, and MD assisted with the design of the study, carried out the intervention, and critically reviewed the manuscript. RS conceived and designed the study, and performed interpretation of data and critical review of the manuscript. All authors read and approved the final manuscript.

## Pre-publication history

The pre-publication history for this paper can be accessed here:

http://www.biomedcentral.com/1471-2431/12/52/prepub
